# Design of a novel antimicrobial peptide 1018M targeted ppGpp to inhibit MRSA biofilm formation

**DOI:** 10.1186/s13568-021-01208-6

**Published:** 2021-03-26

**Authors:** Zhou Jiale, Jiao Jian, Tan Xinyi, Xie Haoji, Huang Xueqin, Wang Xiao

**Affiliations:** 1 Immunology Innovation Team, School of Medicine, Ningbo University, University, 818 Fenghua St., Jiangbei District, Ningbo, 315211 Zhejiang China; 2 Department of biomedicine, Beijing City University, Beijing, 100094 China

**Keywords:** MRSA, Antimicrobial peptide 1018M, Antibacterial, Antibiofilm, PpGpp, Mechanisms

## Abstract

Methicillin-resistant *staphylococcus aureus* (MRSA) and its biofilm infection were considered as one of the main international health issues. There are still many challenges for treatment using traditional antibiotics. In this study, a mutant peptide of innate defense regulator (IDR-)1018 named 1018M was designed based on molecular docking and amino acid substitution technology. The antibacterial/biofilm activity and mechanisms against MRSA of 1018M were investigated for the first time. The minimum inhibitory concentration (MIC) of 1018M was reduced 1 time (MIC = 2 μg/mL) compared to IDR-1018. After treatment with 32 μg/mL 1018M for 24 h, the percentage of biofilm decreased by 78.9%, which was more effective than the parental peptide. The results of mechanisms exploration showed that 1018M was more potent than IDR-1018 at destructing bacterial cell wall, permeating cell membrane (20.4%–50.1% vs 1.45%–10.6%) and binding to stringent response signaling molecule ppGpp (increased 27.9%). Additionally, the peptides could also exert their activity by disrupting genomic DNA, regulating the expression of ppGpp metabolism and biofilm forming related genes (*RSH*, *relP*, *relQ*, *rsbU*, *sigB*, *spA*, *codY*, *agrA* and *icaD*). Moreover, the higher temperature, pH and pepsase stabilities provide 1018M better processing, storage and internal environmental tolerance. These data indicated that 1018M may be a potential candidate peptide for the treatment of MRSA and its biofilm infections.

**Supplementary Information:**

The online version contains supplementary material available at 10.1186/s13568-021-01208-6.

## Key points

- 1018M showed antibacterial/biofilm activity towards MRSA.
- 1018M destructed bacterial cell wall, permeated cell membrane and bound ppGpp.
- 1018M regulated the expression of ppGpp metabolism and biofilm forming related genes.

## Introduction

*Staphylococcus aureus* (*S. aureus*) is an important trigger factor of a great diversity of acute and chronic infections (Lauderdale et al. 2009). The infections are endemic worldwide and caused noteworthy morbidity and mortality. Under the long-term pressure of overuse and abuse of antibiotics, *S. aureus* has developed its own strategy to evolve into multidrug-resistant pathogen for survival (Hong et al. 2016). The challenge of methicillin-resistant *S. aureus* (MRSA) has become an international health issue, and Asia is one of the regions with the highest prevalence. In China, the prevalence rate of MRSA has reached 50 to 70% of total *S. aureus* isolates (Chen and Huang 2014; Liu et al. 2009; Xiao et al. 2011). *S. aureus* could attach to the surfaces of medical implants or host tissues and form biofilm, which is one of the major causes of recalcitrant and chronic infections (Lauderdale et al. 2009). Up to 80% of human bacterial infections are biofilm associated (Romling and Balsalobre 2012). The self-produced extracellular matrix can help MRSA biofilm evade host immune response and antibacterial drugs (Jolivet-Gougeon and Bonnaure-Mallet 2014; Mansour et al. 2016). Biofilm-encased bacteria are 100–1000 times more tolerant to conventional antibiotics than corresponding planktonic cells (Flemming and Wingender 2010). Therefore, it is urgent to find new effective drugs to treat MRSA and its biofilm infection.

The stringent response signaling molecule ppGpp is an important signal in biofilm development (de la Fuente-Nunez et al. 2014). It serves as a second messenger that is induced by a variety of stress conditions. It has been demonstrated that nutrient starvation is a common condition in biofilm subpopulations, which could increase ppGpp level and cause tolerance to antibiotic exposure. ppGpp is able to regulate the expression of a plethora of biofilm formation relevant genes (Potrykus and Cashel 2008), such as regulators of exopolysaccharides (EPSs) and adhesin genes, then mediate the synthesis of biofilm matrix and control the biofilm formation and pellicle structure (Nielsen et al. 2011). The majority of studies suggested that a clear reduction in biofilm formation is detected in the absence of ppGpp (Romling and Balsalobre 2012). Therefore, targeting ppGpp to prevent the formation of biofilm has become a novel approach for potential antimicrobial and anti-biofilm strategies (Syal et al. 2017).

Antimicrobial peptides (AMPs) are kind of bioactive agents with multiple modes of action and appear as one of the most promising antimicrobial drug candidates (Hancock and Sahl 2006). It has been demonstrated that AMPs have broad-spectrum efficacy in combatting drug-resistant bacteria and biofilms (Pletzer and Hancock 2016; Sanchez-Gomez and Martinez-de-Tejada 2017). The antimicrobial activity of AMPs was mainly based on their positive charge and hydrophobic residues, which gave them the properties of bacterial cell membrane destruction (Mahlapuu et al. 2016). Among them, a small synthetic peptide termed innate defense regulator (IDR-)1018, which was derived by substantial modification of the bovine neutrophil host defense peptide bactenecin, captured our attention (Mansour et al. 2015). As the most potent IDR that has been described, IDR-1018 can prevent the accumulation and accelerate the degradation of ppGpp via direct interaction with this signaling molecule (Rivas-Santiago et al. 2013; Wang et al. 2018). Based on this mechanism, IDR-1018 acquired broad anti-biofilm activity. However, its antibacterial (MIC = 8–256 μg/mL) and anti-biofilm (MBIC_100_ = 2–10 μg/mL) effects remain to be promoted further (de la Fuente-Nunez et al. 2014). Given the above conclusions, we hypothesized that enhancing the binding ability of IDR-1018 with ppGpp and adjusting antimicrobial-related key amino acid residues can achieve the purpose of improving the antibacterial/biofilm activity of this peptide.

Therefore, in this study, the binding sites of IDR-1018 and ppGpp were predicted by molecular docking method, and the novel AMP 1018M was obtained by amino acid substitution. Subsequently, the binding affinity, antibacterial activity, stability, biological characteristics and mechanism of antimicrobial/biofilm activity of 1018M was explored.

## Materials and methods

### Bacterial strains and cell lines

The methicillin-resistant *S. aureus* (MRSA) ATCC43300, methicillin-sensitive *S. aureus* (MSSA) ATCC25923 and ATCC6538 were purchased from the American Type Culture Collection (ATCC). The RAW 264.7 cells were donated by Dr. Guo Hua (Ningbo University).

### 1018M design and preparation

Three-dimensional (3D) structure of IDR-1018 was simulated and analyzed using online tool I-TASSER (Roy et al. 2010; Yang et al. 2015; Yang and Zhang 2015). Then the IDR-1018 (ligand) was docked onto ppGpp (receptor) by AutoDock 4.2 (Kushibiki et al. 2014). Briefly, ppGpp was kept rigid, and all side chains of IDR-1018 were defined as flexible. Grid map of ppGpp was 70 × 80 × 80 points, with a grid spacing of 0.375 Å. Dock was generated by Lamarchian genetic algorithm (LGA) with default parameters. The conformation with minimum binding energy was selected and analyzed.

Based on IDR-1018 as the parental peptide, 1018M was finally designed by substituting amino acid residues (the amino acid residues I4, V5, A6, V7 and I9 were replaced by R, W, W, R and R), which retained the key binding amino acids, increased the positive charge and membrane anchor amino acids appropriately. The 3D structures and binding affinity to ppGpp of 1018M were analyzed by online tool I-TASSER and Autodock software, respectively. The physicochemical parameters of IDR-1018 and 1018M, including molecular weight (MW), isoelectric point (pI), positive charge and grand average of hydropathicity (GRAVY) were predicted by online tool ProtParam (http://web.expasy.org/protparam/). The hydrophobicity was analyzed with online tool HELIQUEST. Hydrophobic ratio was calculated by APD3, an antimicrobial peptide calculator and predictor (http://aps.unmc.edu/AP/prediction/ prediction_ main.php). IDR-1018 and 1018M were synthesized by solid-phase synthesis.

### ppGpp binding affinity

The ppGpp binding affinity of peptides were tested on a ZIC-HILIC PEEK high performance liquid chromatography (HPLC) column (250 × 4.6 mm, 5 μm, Merck Millipore) using the mobile phase consisting of 25 mM ammonium acetate (pH 6.8)/acetonitrile (v/v = 80:20) at a flow rate of 0.5 mL/min and UV detector at 260 nm. The temperature of the column is kept at 25 ℃. The ppGpp (TriLink BioTechnologies, USA) which diluted with mobile phase to a concentration of 1000 μg/mL, was used as a positive control. Then the ppGpp (1000 μg/mL) was incubated with peptides (400 μg/mL) for 10 min and centrifuged at 12000 rpm for 10 min. The ppGpp concentration of the samples was calculated by comparing the peak areas of the ppGpp standard. All assays were performed in triplicate (Xu et al. 2016).

### Antimicrobial activity

#### Minimum inhibitory concentration (MIC)

The MIC values of peptides against test stains were determined by the microtiter broth dilution method. The bacteria were grown to mid-logarithmic phase and diluted to 1 × 10^5^ CFU/mL. Two-fold serial dilutions of peptides were prepared with a gradient concentration of 1280, 640, 320, 160, 80, 40, 20, 10, 5, 2.5, 1.25, and 0.625 μg/mL. A total of 10 μL each serial concentration gradient peptide solutions and 90 μL bacterial suspension were added into 96-well plates. Then the plate was cultured at 37 ℃ for 16–24 h. Vancomycin was tested as control. All assays were performed in triplicate. The MIC refers to the lowest peptide concentration at which there is no visible growth of bacteria (Wiegand et al. 2008).

#### Time-kill curves

The time-kill curves against MRSA ATCC43300 were assayed to evaluate the pharmacodynamics of peptides. The mid-log phase bacteria were diluted to 1×10^5^ CFU/mL with fresh medium. Subsequently, MRSA ATCC43300 bacteria solution (5 mL) and various concentrations of peptides (final concentration 1×, 2×, 4× MIC) were added to a 50-mL shaking flask. Finally, the mixtures were cultured at 37 °C and 250 rpm. Samples (150 μL) were taken from each flask at 0, 0.5, 1, 2, 4, 6, 8, 10, 12 and 24 h of incubation, and antimicrobial efficiency was measured by plate colony count (Yang et al. 2019). Treatment with peptide solvent (PBS) and vancomycin at 2× MIC were used as the negative and positive control. All tests were run in triplicate.

### Toxicity and stability

#### Hemolysis

Hemolytic activity was evaluated by determining hemoglobin released from healthy mouse red blood cells (Yang et al. 2017). Blood cells were washed three times in sterile PBS (10 mM, pH 7.4) and centrifuged at 1500 rpm for 5 min at room temperature. An amount of 100 μL red blood suspensions (8%, v/v) were mixed with 100 μL peptides at different concentrations (0.25–128 μg/mL). Then the mixtures were incubated at 37 °C for 1 h and centrifuged at 1500 rpm for 5 min. Finally, absorbance of supernatants was measured at 540 nm. PBS and 0.1% Triton X-100 served as negative and positive controls. Three replicates were performed for each condition. Hemolysis = [(OD540 nm of the treated sample-OD540 nm of the negative control)/(OD540 nm of positive control-OD540 nm of negative control)] × 100%.

#### Cytotoxicity

To determine the effect of peptides on the viability of murine peritoneal RAW264.7 macrophage cells, Cell Counting Kit-8 (CCK-8) assay was performed according to a previous method (An and Cheng 2020). Cells (2.5 × 10^4^ cells/well) were added into 96-well plates and incubated in a humidified 5% CO_2_ environment at 37 °C overnight. The cells were then incubated with various concentrations (0.5–128 μg/mL) of peptides for 24 h. Equal volume PBS treated cells were used as control. WST-8 solution (10 µL) was added to each well followed by incubating for 4 h at 37 ºC. A microplate reader was used to measure the absorbance at 460 nm. Three replicates were performed for each condition. The cell viability was calculated using the following formula: Cell viability = OD 460 nm of treated sample/OD 460 nm of control × 100%.

#### Temperature, protease and pH stability

To analyze the thermal stability of peptides, IDR-1018 and 1018M were incubated at different temperatures (4, 20, 40, 60, 80, and 100 °C) for 1 h. The pH stability of peptides was determined after 3 h incubation in 100 mM glycine-HCl buffer (pH 2.0), sodium acetate buffer (pH 4.0), sodium phosphate buffer (pH 6.0), Tris-HCl buffer (pH 8.0), or glycine-NaOH buffer (pH 10.0). Additionally, peptides were incubated in pepsin (3000 U/mg, pH 2.0) and trypsin (250 U/mg, pH 8.0) (10:1, w/w) solutions for 4 h at 37 °C, respectively. The untreated peptides were used as control. The antibacterial activity of treated peptides against MRSA ATCC43300 was determined by MIC determine assay (Yang et al. 2017). All assays were performed in triplicate.

### Effects of 1018M on cell wall and membrane

#### Scanning/transmission electron (SEM/TEM) microscope observations

Mid-log phase MRSA ATCC43300 (1 × 10^8^ CFU/mL) were treated with 4× MIC IDR-1018 and 1018M for 2 h at 37 °C. The bacteria were fixed with 2.5% glutaraldehyde at 4 °C overnight. For SEM, the bacterial were then dehydrated with a series of graded ethanol solutions (20%, 50%, 70%, 85%, 95% and 100%) and dried by CO_2_. Gold-palladium was sputtered on samples and observed on a S4800 SEM. For TEM, bacterial were post-fixed with 1% OsO4 for 1 h and then dehydrated for 7 min each time with a graded acetone series (50%, 70%, 85%, 95% and 100%). The samples were immersed in epoxy resin and embedded in capsules containing embedding medium, polymerized at 45 °C for 3 h and at 65 °C for 24 h, respectively. Ultramicrotome was used to acquire thin sections, followed by staining with 1% uranyl acetate. Images were visualized by a Hitachi H-7650 TEM (Yang et al. 2019).

#### Membrane permeabilization analysis

The bacterial cell membrane permeabilization activity of peptides was investigated by the propidium iodide (PI) uptake assay (Wang et al. 2016). Mid-log phase MRSA ATCC43300 (1 × 10^8^ CFU/mL) were incubated with or without 1× MIC, 2× MIC and 4× MIC peptide solutions at 37 °C for 30 and 120 min, respectively. Bacteria were washed twice with PBS, incubated with 50 μg/mL PI for 15 min. Finally, the fluorescence was analyzed by FACS Calibur Flow Cytometer (BD, USA) (Yang et al. 2017).

### Effects of 1018M on bacterial genomic DNA

#### Gel retardation assay

To examine the interaction of peptides and MRSA genomic DNA, the gel migration experiment was performed. Genomic DNA was obtained by bacterial genome extraction kit. Different concentrations (0.625 to 20 μg/mL) of peptides and DNA were mixed and incubated for 10 min at room temperature. The migration of genomic DNA was analyzed by electrophoresis on a 1% agarose gel.

#### Circular dichroism (CD) spectroscopy

To examine the secondary structure changes of MRSA ATCC43300 genomic DNA after treatment with peptides, the CD spectra were measured as a previously described method (Wang et al. 2017). Peptides (40 μg/mL) and genomic DNA (150 μg/mL) were mixed and incubated for 10 min at room temperature. Then the samples were loaded into a cuvette with 1.0-mm path length and followed by testing on a CD spectrometer (J-1700 CD). The spectra were recorded from 220 to 320 nm at 25 °C with a 10 nm/min scanning speed.

### Ability of 1018M against MRSA biofilms

#### Effects on biofilm formation

To evaluate the effect of peptides on the biofilm information, mid-logarithmic phase MRSA ATCC43300 (1×10^8^ CFU/mL) was grown in tryptic soy broth (TSB) medium on 96-well plates at 37 °C for  h in the presence (2–64 μg/mL) or absence of peptides (de Breij et al. 2018; de Breij et al. 2016). Subsequently, the planktonic bacteria were removed and biofilms were stained for 30 min with 0.1% crystal violet. After rinsing with PBS twice, the dye binding to the adherent bacterial cells was resolubilized in 95% ethanol (200 μL/well). Finally, absorbance was measured at 570 nm with a microplate reader. Fresh TSB medium was used as negative control.

#### Biofilms observed by confocal laser scanning microscopy (CLSM) and SEM

In order to further explore whether the formation of MRSA ATCC43300 biofilms can be inhibited by peptides, the mid-log phase bacteria were diluted to 1 × 10^8^ CFU/mL with TSB medium and seeded into 24-well plates with a glass microscope slide or silicon slice in each well. A concentration of 64 μg/mL peptides were added into plates and incubated for 24 h. Planktonic bacteria were removed by washing with PBS gently. Biofilms on glass microscope slide were stained with SYTO9 and PI according to the instruction (LIVE/DEAD BacLight Bacterial Viability Kit). After incubation for 15 min, the slides were washed with PBS and the biofilms were visualized by SP8 CLSM. Biofilm on silicon slice could be observed by SEM after immobilization, dehydration, drying and coating (Yang et al. 2019).

### Effects of peptides on transcription of ppGpp metabolism and biofilm formation related genes

The ppGpp metabolism and biofilm formation related genes *RSH*, *relP*, *relQ*, *rsbU*, *sigB*, *spA*, *codY*, *agrA* and *icaD* were chosen in this study. Primer sequences were designed and listed in Additional file 1: Table S1. The 16s rRNA was used as a housekeeping one. The test bacteria were treated with 4× MIC peptides for 2 h. After washing twice with PBS, the total RNA was isolated. Then cDNA was obtained followed by removing genomic DNA. Finally, real-time reverse transcription-polymerase chain reaction (qRT-PCR) was conducted as previously described. The relative expression ratios were calculated as follows: n-fold transcription = 2^−△△Ct^, ∆∆Ct = ∆Ct (drug-treated)/∆Ct (untreated), where ∆Ct represents the difference between the cycle threshold (Ct) of the gene studied and the Ct of housekeeping 16s rRNA gene (internal control). Student’s t test was used for analyzing those PCR results (Lee et al. 2013).

### Statistical analysis

All data were analyzed using GraphPad Prism 6 and the results are presented as the mean ± SD (standard error of the mean). Comparisons among multiple groups were performed by one-way ANOVA or student’s t test. A p-value of < 0.05 was considered statistically significant.

## Results

### 1018M design and preparation

**Fig. 1:**
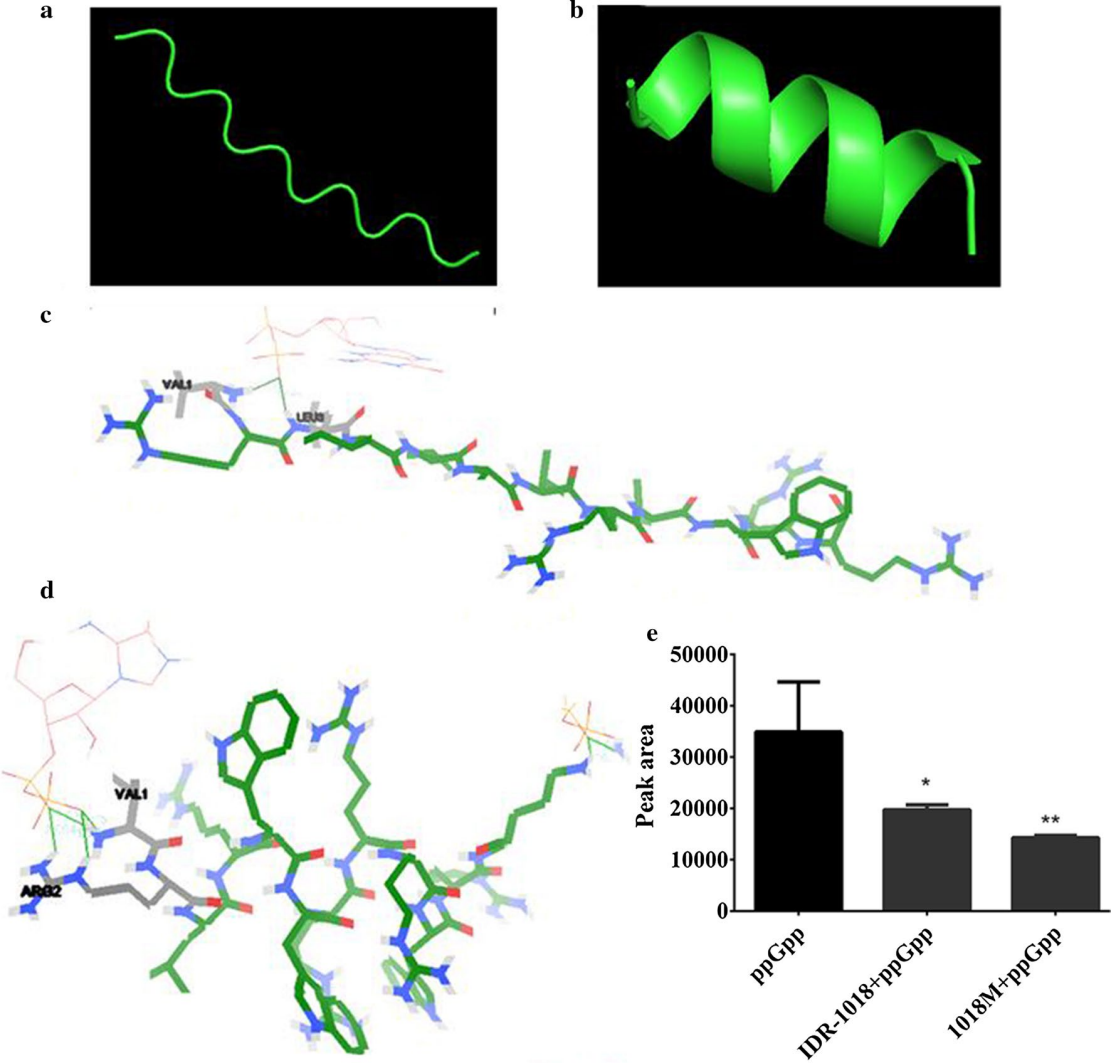
Predictions of 1018M tertiary structures and binding affinity of 1018M with ppGpp. **a**, **b** Predictions of IDR-1018 (**a**) and 1018M (**b**) tertiary structures. **c**, **d** Molecular docking of IDR-1018 (**c**) and 1018M (**d**) with ppGpp. **e** Binding affinity of IDR-1018 and 1018M with ppGpp

The predictions of peptides’ tertiary structure were shown in Fig. 1a, b and Additional file 1: Fig. S1. IDR-1018 adopted a random coil structure (Fig. 1a). Molecular docking result exhibited that IDR-1018 bound to ppGpp molecules by N terminal (Fig. 1c) and formed two hydrogen bonds through Val1 and Leu3 amino acids. The binding energy was − 3.52, KI value was 261.00 μM, and the lengths of two hydrogen bonds were 1.779 and 1.798 nm respectively. Therefore, we substituted C terminal amino acid residues to improve the positive charge and membrane anchor ability. As shown in Fig. 1b, 1018M presents a α-helix structure. As anticipated, the net charge and isoelectric point (pI) of 1018M were obviously higher than IDR-1018 (7 and 4, 12.78 and 12.48, respectively). However, GRAVY, hydrophobicity and hydrophobic ratio of novel peptide were lower than that of parental peptide (0.692 and − 2.183, 0.623 and 0.217, 66% and 41%, respectively) (Table 1). Interestingly, the binding ability to ppGpp of 1018M was promoted by the novel α-helix structure. 1018M formed four hydrogen bonds with ppGpp through two amino acids Val1 and Arg2 (Fig. 1d) with a binding energy of − 4.62, a KI value of 410.62 μM and four hydrogen bonds of 1.630, 2.054, 1.715, 1.944 nm severally. Compared with IDR-1018, 1018M increased the capacity of binding ppGpp by about 57% (calculated by KI values).

**Table 1:** The sequences and physicochemical parameters of IDR-1018 and 1018M

Name	Sequence	MW(Da)	pI	Charge (+)	GRAVY	Hydrophobicity	Hydrophobic ratio
IDR-1018	VRLIVAVRIWRR-NH2	1536.93	12.48	4	0.692	0.623	66%
1018M	VRLRWWRRRWRR-NH2	1882.26	12.78	7	− 2.183	0.217	41%

### ppGpp binding affinity

To determine the ppGpp binding affinity of peptides, the HPLC was used to measure the peptide and ppGpp mixture samples. The concrete data and statistical results of HPLC were shown in Additional file 1: Fig. S2 and Fig. 1e, which indicated that the ppGpp concentrations after incubation with IDR-1018 (mean peak areas were 19723 and 14255, respectively) were significantly lower than control group (peak area was 34904) (Fig. 1e, Additional file 1: Fig. S2). According to the peak areas data, we noted that the binding affinity of 1018M to ppGpp was increased by 27.9%, which was consistent with the predicted conclusion.

### Antimicrobial activity

#### MIC determination

As shown in Table 2, MIC of IDR-1018 against MRSA ATCC43300, MSSA ATCC25923 and MSSA ATCC6538 was 4, 4 and 2 μg/mL, respectively. Compared to parental peptide, 1018M displayed more potent antibacterial activity against these bacteria with MIC value of 2, 2 and 1 μg/mL, respectively. However, the antimicrobial activity of 1018M was not as good as that of vancomycin (MIC = 1 μg/mL).

**Table 2:** The MICs of IDR-1018, 1018M and vancomycin against *S. aureus*

Drugs	MICs (μg/mL)		
	ATCC43300	ATCC25923	ATCC6538
IDR-1018	4	4	2
1018M	2	2	1
vancomycin	1	1	1

#### Time-killing curves

**Fig. 2:**
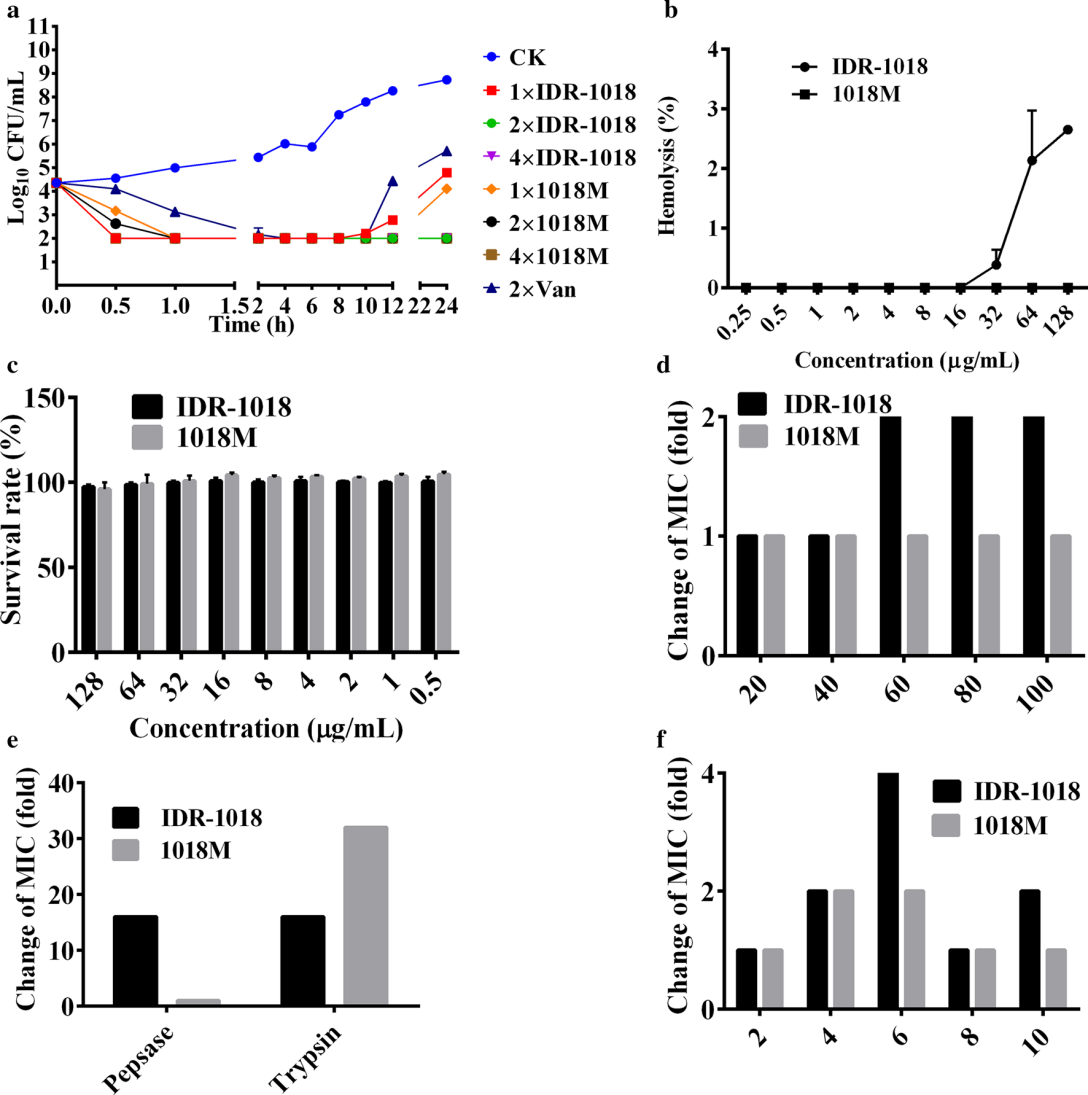
The extracellular killing curves, toxicity and stability of 1018M. **a** Bactericidal kinetics assay of MRSA ATCC43300 treated with IDR-1018, 1018M and Vancomycin. **b** The hemolyses of 1018 and 1018M against the red blood cells of mice. **c** The cytotoxicities of 1018 and 1018M against the RAW 264.7 cells. **d**–**f** The temperature (**d**), protease (**e**) and pH (**f**) stability of IDR-1018 and 1018M

As a representative strain, MRSA ATCC43300 was used for subsequent tests. Time-killing kinetic curves (Fig. 2a) showed that after treatment with 1×, 2×, and 4× MIC of IDR-1018 and 1018M, the bacteria amount of MRSA ATCC43300 was significantly decreased within 0.5 h in a time- and concentration-dependent manner. The antimicrobial efficiency of peptides was superior to vancomycin, which exhibited the slowest antibacterial rate within 4 h. Furthermore, the bacteria rejuvenated after incubation with 2× MIC vancomycin for 10 h. However, the antibacterial effect of 2× and 4× MIC peptides could last for 24 h. When compared the novel and parental peptide, we found that IDR-1018 with fast bactericidal activity in the early stage, while the effect of 1018M was better in the late stage.

### Toxicity and stability

#### Hemolysis

The toxicity of peptides to eukaryotic cells was determined by its ability to lyse murine erythrocytes. Hemolytic activities of IDR-1018 were 0.386%, 2.136% and 2.651% at the concentrations of 32, 64 and 128 μg/mL, respectively. However, hemolysis of 1018M was 0% at all tested concentrations, which was obviously lower than IDR-1018 (Fig. 2b). The results indicated that 1018M does not impair the integrity of red blood cells at effective concentrations and is a potential candidate mainline drug against bacterial infection.

#### Cytotoxicity

The cytotoxicity of IDR-1018 and 1018M in murine RAW264.7 macrophage cells was determined via the CCK-8 assay. As shown in Fig. 2c, the survival rate of IDR-1018 and 1018M at a concentration of 128 μg/mL was 97.3% and 95.9%, respectively, indicating that the two peptides have very low cytotoxicity activity against murine macrophage cells.

#### Temperature, protease and pH stability

The temperature, protease and pH stability of IDR-1018 and 1018M were presented in Fig. 2d–f. After exposure to 20, 40, 60, 80 or 100 °C temperatures for 1 h, the antibacterial activity of 1018M was not changed. While the parental peptide IDR-1018 had potent antibacterial activity against MRSA ATCC43300 after treatment at 20 or 40 °C, but retained only 50% activity at 100 °C. Moreover, 1018M also showed higher stability after treatment with pepsase (100% activity) than IDR-1018 (MIC = 64 μg/mL) (Fig. 2e). However, both IDR-1018 and 1018M could not tolerant the degradation of trypsin, the MICs were 16-32 times of the untreated peptides. Furthermore, the MIC values of 1018M at pH 2–10 were 2–4 μg/mL, which were 1–2 folds of the normal condition. Compared to the novel peptide, IDR-1018 was slightly unstable with 1-4 times MIC of the MH medium.

### Effects of 1018M on cell wall and membrane

#### Scanning/transmission electron (SEM/TEM) microscope observations

**Fig. 3:**
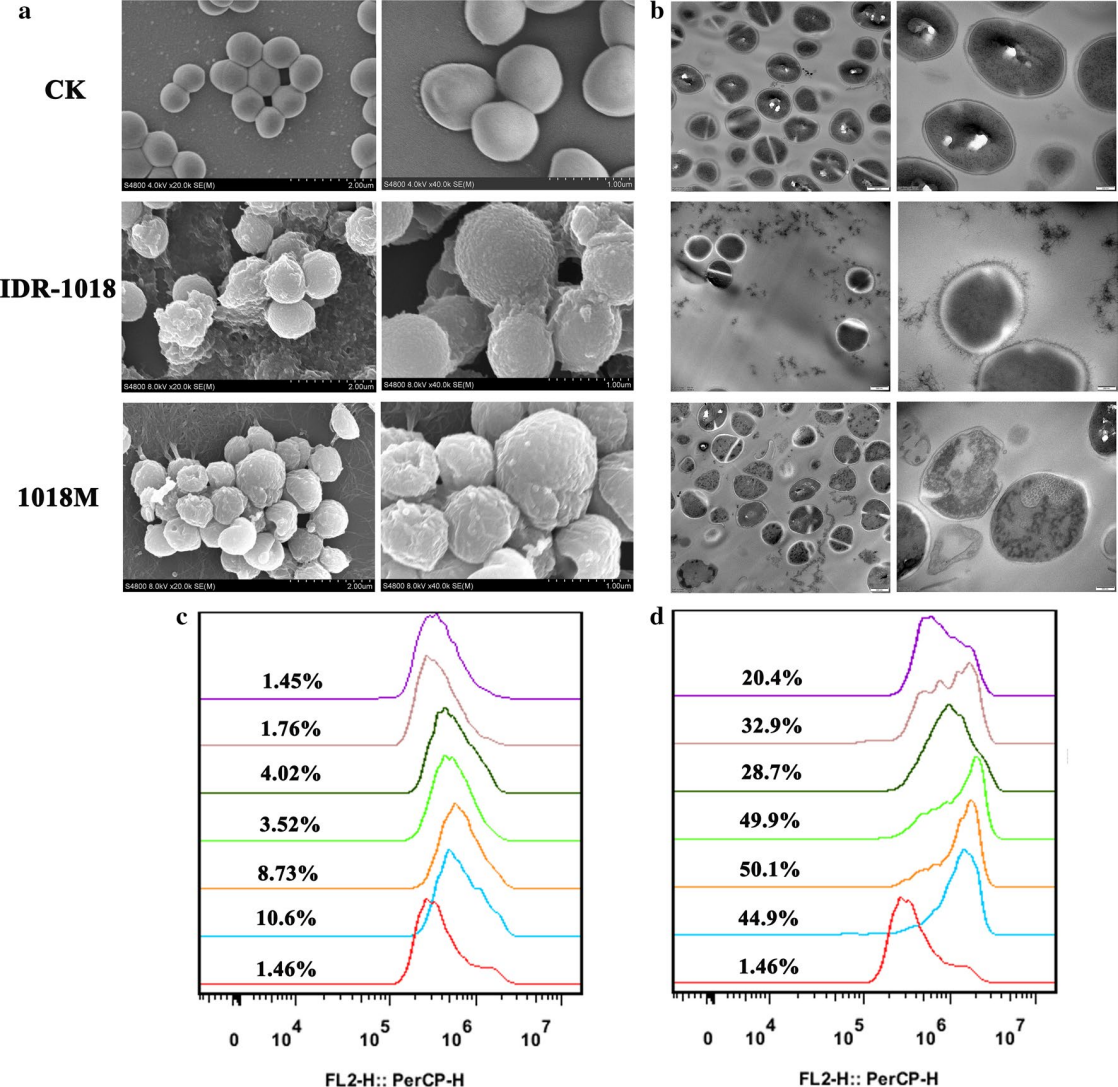
Effects of 1018M on cell wall and membrane. **a–b** Scanning electron microscopy (**a**) and transmission electron microscopy (**b**) analysis of MRSA ATCC43300 cells treated with IDR-1018 and 1018M. **c**–**d** Flow cytometric analysis of PI-staining in MRSA ATCC43300 cells treated with 2× or 4× MIC IDR-1018 (**c**) and 1018M (**d**) for 30 or 120 min, respectively. Red line: no peptide, negative control; Blue line: treatment with 1×MIC peptides for 30 min; Orange line: treatment with 2×MIC peptides for 30 min; Green line: treatment with 4×MIC peptides for 30 min. black line: treatment with 1×MIC peptides for 120 min; brown line: treatment with 2×MIC peptides for 120 min; purple line: treatment with 4×MIC peptides for 120 min

The change in bacteria morphology, integrity, and cellular structure after treatment with peptides could be observed by SEM. As shown in Fig. 3a, the untreated MRSA ATCC43300 bacteria exhibited normal morphologies and smooth surfaces. After treatment with 4× MIC IDR-1018, over 50% shrunken, bubbling bulges and filiferous adhesive substances bacteria were observed in the image. For the 1018M group, nearly 100% of the same changes bacteria were found in the image and the destructions were more serious, holes or disruptions were found in the bacterial surface. Additionally, the internal ultrastructures were observed by TEM (Fig. 3b). The ultrastructures were normal and cytoplasm appeared to have homogeneous electron density in control group. After exposure to IDR-1018, only mild bacterial cell wall damage was observed. However, treatment with 1018M caused severe damage to bacterial cell walls, cell membranes and cytoplasm. About 10% ghost bacteria were found in the image. Deformed cell morphology and cellular contents leakage were observed. Cytoplasm displayed a heterogeneous electron density, indicating a binding action of 1018M to the intracellular material.

#### Membrane permeabilization analysis

The nucleic acid fluorescent dye PI can penetrate the damaged cell membranes. Therefore, in this study, it was used to evaluate the effects of peptides on the bacterial cell wall and membrane of MRSA ATCC43300 by flow cytometry. In the untreated group, the fluorescence rate of MRSA ATCC43300 was 1.46%, which indicated that bacteria had intact cell membranes. After treatment with 1×, 2×, and 4× MIC 1018M for 0.5 h and 2 h, the percentages of PI-permeable cells were 20.4%–50.1%, higher than those of IDR-1018 (1.45%–10.6%) (Figs. 3c and d), indicating the stronger penetrating ability of 1018M.

### Effects of 1018M on bacterial genomic DNA

#### Gel retardation assay

**Fig. 4:**
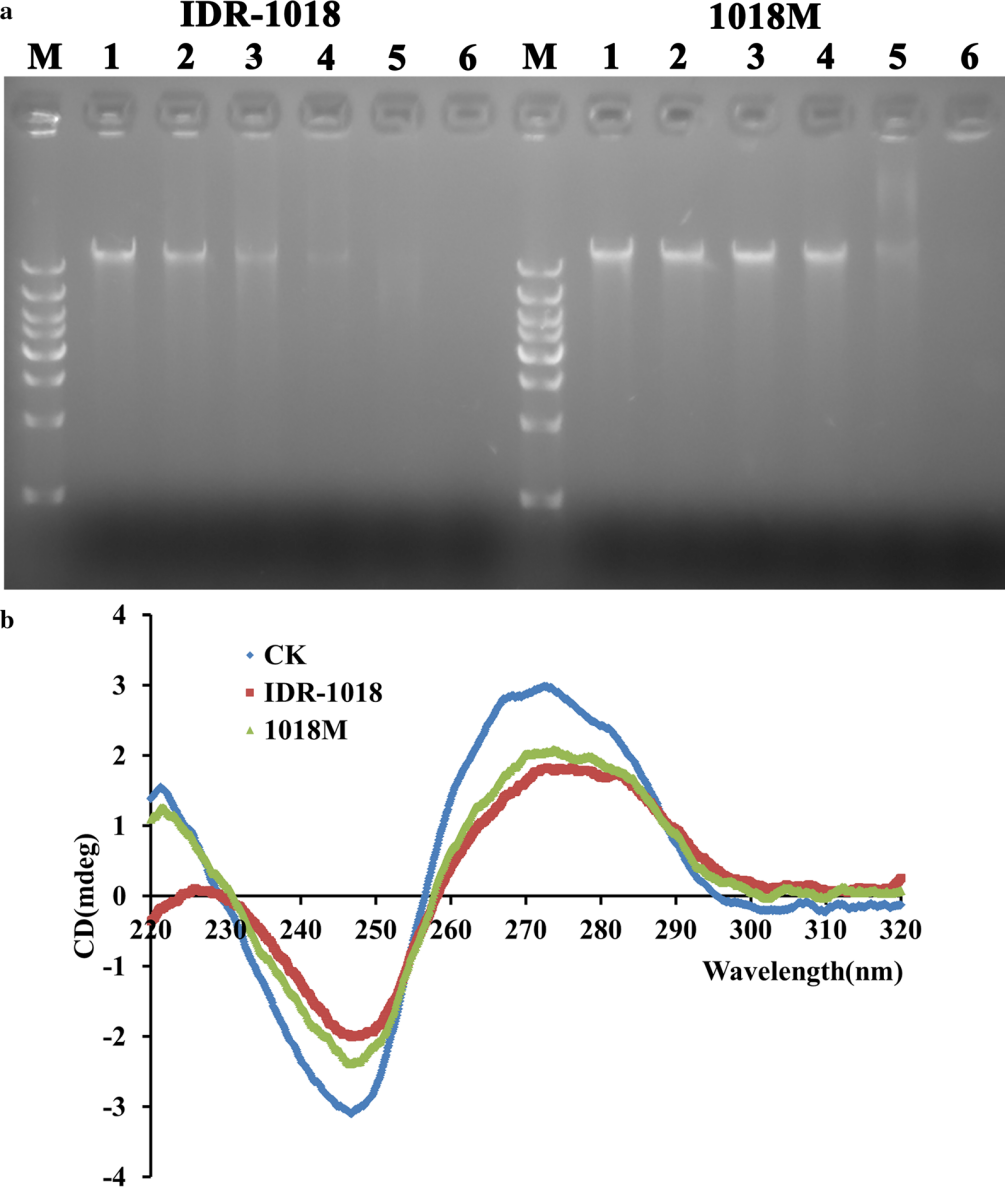
Interaction of 1018M with MRSA ATCC43300 bacterial genomic DNA. **a** Interaction of IDR-1018 and 1018M with bacterial genomic DNA by a gel migration assay. M: DNA marker; 1–6: The concentration of peptides were 0, 0.625, 1.25, 2.5, 5, 10 μg/mL, respectively. **b** CD spectra of genomic DNA from MRSA ATCC43300 in the presence of IDR-1018 and 1018M. The concentration of peptide and DNA were 40 and 150 μg/mL, respectively

To evaluate the DNA-binding properties of peptides, the DNA gel movement retardation assay was performed. IDR-1018 and 1018M inhibited the migration of genomic DNA from MRSA ATCC43300 at the concentration of 1.25 and 5 μg/mL, respectively (Fig. 4a). This result indicated that IDR-1018 and 1018M can bind to bacterial genomic DNA, and the binding efficiency of novel peptide was lower than that of parental peptide.

#### CD spectroscopy

The affinity of IDR-1018 and 1018M binding to DNA were further detected using a CD spectrometer, which was usually used to monitor changes in DNA morphology when drugs interact with DNA. The positive and negative peak of MRSA ATCC43300 genomic DNA appeared at about 270 and 245 nm in the CD spectrum (Fig. 4b). After treatment with IDR-1018 or 1018M, the DNA ellipticity intensity decreased, indicating that IDR-1018 and 1018M maybe bind to MRSA ATCC43300 genomic DNA and changed the DNA conformation. The binding affinity of IDR-1018 was slightly higher than 1018M, which was inconsistent with gel retardation assay.

### Ability of 1018M against MRSA biofilms

#### Inhibition of biofilm formation

**Fig. 5:**
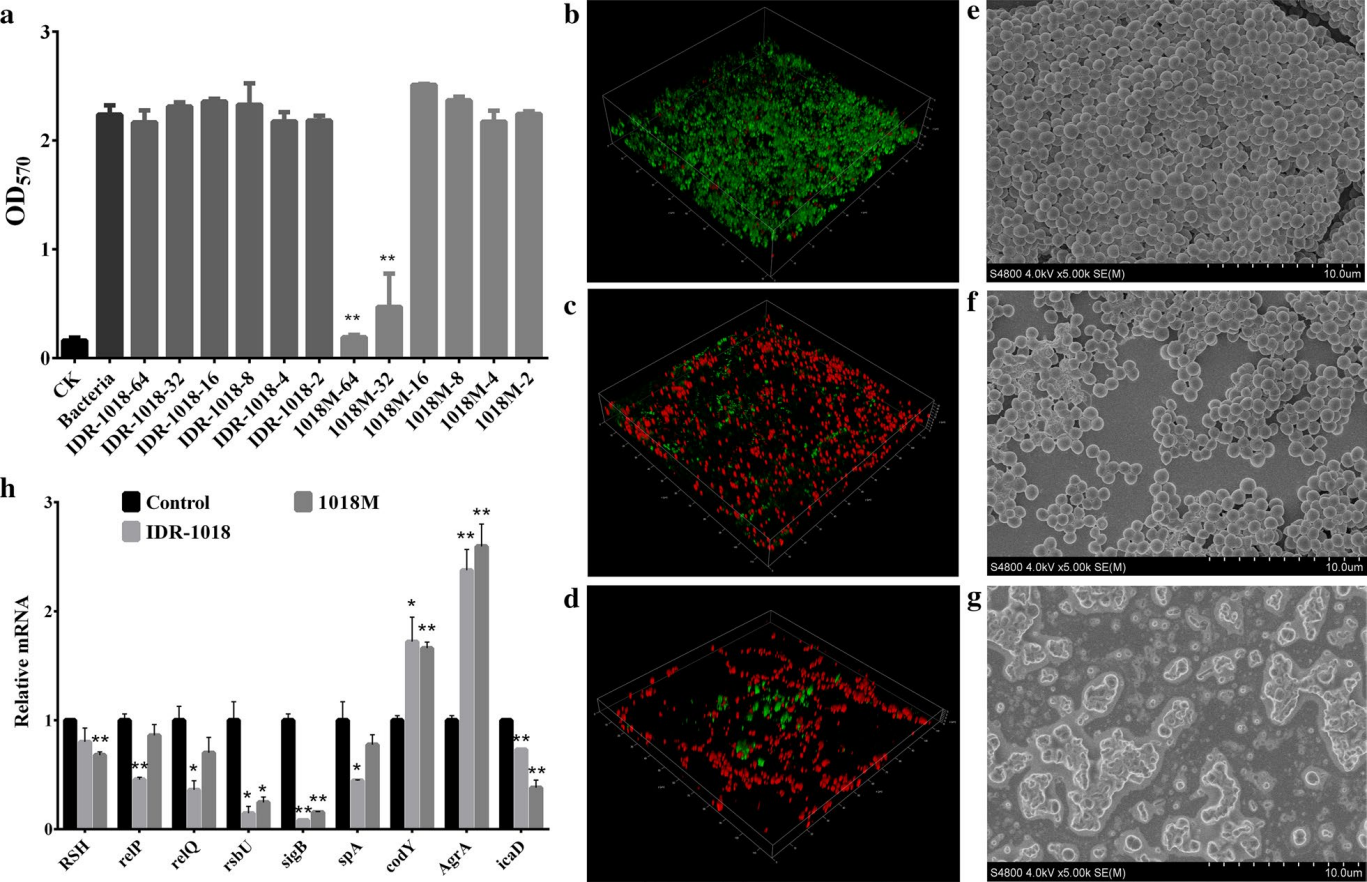
The abilities of 1018 and 1018M against MRSA biofilms. **a** Inhibition effect of IDR-1018 and 1018M on biofilms formation. **b**–**d** Observation of MRSA ATCC43300 biofilms by CLSM. MRSA ATCC43300 was incubated with 64 μg/mL IDR-1018 (**c**), 1018M (**d**) for 24 h, and biofilms were stained with dyes and visualized by CLSM after removing planktonic bacteria. Live cells are stained in green by SYTO9 and dead cells are stained in red by PI. **b** The untreated biofilm. **e**–**g** Observation of MRSA ATCC43300 biofilms by SEM. **e** Untreated biofilm, **f** IDR-1018, **g** 1018M. **h** Relative gene expressions of biofilms. MRSA ATCC43300 cells were incubated with 4× MIC peptides or no peptide for 2 h and collected by centrifugation. After isolation RNA from cells, the transcriptional levels of ppGpp metabolism and biofilm formation related genes were detected by qRT-PCR. All assays were performed in triplicate. The analyses were measured by one-way ANOVA, with Duncan’s multiple comparisons test. A probability value of < 0.05 was considered significant. (*) Indicates the significance between control and each of treatment groups. * p < 0.05; ** p < 0. 01. The results are given as the mean ± SD (n = 3)

As shown in Fig. 5a, 1018M inhibited MRSA ATCC43300 biofilm formation in a concentration-dependent manner. After treatment with 32 and 64 μg/mL of 1018M for 24 h, the percentages of biofilm decreased by 91.5% and 78.9%, which were significantly lower than control group. However, IDR-1018 had nearly no impact on biofilm formation, indicating that the inhibition efficiency of 1018M against MRSA ATCC43300 biofilm was remarkably higher than that of IDR-1018.

#### Inhibition of biofilms observed by CLSM and SEM

In order to further confirm the biofilm formation inhibition activity of 1018M, the 64 μg/mL peptide treated MRSA ATCC43300 was observed by CLSM. As shown in Fig. 5b–d, the control group bacteria formed thick biofilms on the surfaces of the glass plates and most of the bacteria were alive. Conversely, 1018M treatment caused a sharp decline in the number of live bacteria and completely inhibited the biofilm formation. We observed the same phenomenon in the SEM photograph. IDR-1018 showed a weaker adhesion of the bacteria and over 90% of the bacteria presented normal morphologies. For the 1018M group, the attached bacteria were all destroyed by peptide (Fig. 5e–g). All the results indicated that the anti-biofilm activity of 1018M is superior to IDR-1018.

### Effects of peptides on transcription of ppGpp metabolism and biofilm formation related genes

To evaluate the mRNA transcription levels of MRSA ATCC43300 ppGpp metabolism and biofilm formation related genes, bacteria cells were treated with 4×MIC peptides for 2 h. The results showed in Fig. 5h indicated that the mRNA transcription of *RSH*, *relP*, *relQ*, *rsbU*, *sigB*, *spA* and *icaD* genes were significantly decreased (0.08-0.86 fold to the control level). The transcription of *codY* and *AgrA* genes in IDR-1018 and 1018M treated groups were 1.72, 2.375 and 1.66, 2.595 time upregulated, respectively. These observations suggested that IDR-1018 and its derivative 1018M inhibited the formation of biofilms mainly by regulating the expression of the related genes in MRSA ATCC43300.

## Discussion

MRSA infection is still a major global healthcare problem, which exhibits high rates of morbidity and mortality. Resistance to first-line antimicrobials combined with a lack of equally effective alternatives complicates MRSA bacteremia treatment (Hassoun et al. 2017). Additionally, *S. aureus* is a leading cause of biofilm infections, which cause significant problems in the chronic hospital infections (Periasamy et al. 2012). In this study, based on a potent antimicrobial peptide IDR-1018 that worked by blocking biofilm development signal molecular ppGpp (Davies 2003), we designed a novel peptide 1018M by substituting amino acids to improve the positive charge, ppGpp binding and membrane anchor ability, and followed by the determination of its antibacterial and anti-biofilm activity.

Though previous results have confirmed that peptide IDR-1018 could trigger the degradation of (p)ppGpp signal molecular within bacteria and act as a broad-spectrum biofilm inhibitor (de la Fuente-Nunez et al. 2014), the antimicrobial and anti-biofilm activity of IDR-1018 still need to be improved. Therefore, the 3D structure and binding position with ppGpp of the peptide were predicted. The result showed that amino acids in N terminal (Val1 and Leu3) played an important role in molecular interactions (Fig. 1). Hence, C terminal amino acids became the targets for modification.

For replacement site selection, it has been proved that Ile-4 of IDR-1018 could be substituted with large hydrophobic residues as well as Gly and Arg, Val-5 and Val-7 could also be replaced by large hydrophobic residues without reducing the peptide’s anti-biofilm activity. All substitutions at Ala-6 were well tolerated except for Gly. The positions of Ile-9 allowed slightly more diverse amino acid substitutions (Haney et al. 2018). For substitution amino acid residues selection, most antimicrobial peptides are rich in Arg and Trp residues which possess some vital chemical properties to guarantee the efficiency of peptides. Trp has an evident preference for the interfacial region of lipid bilayers. Additionally, the folding of peptides in aqueous solution is also related to Trp sidechains, which can keep the native and normative hydrophobic contacts. Arg provides cationic charges and hydrogen bonding properties for the peptide, thus leading to an interaction with the abundant anionic components of bacterial membranes. In general, these two residues could maintain the conformation of the peptide, facilitate the peptide-membrane interactions by participating in cation-pi interactions, and even lead to structures for membrane-mimetic bound peptides, then endow the peptide with high activity even at very short lengths (Chan et al. 2006). Therefore, in our study, the amino acid residues I4, V5, A6, V7 and I9 were replaced by R, W, W, R and R to enhance the content of Trp and Arg, thus leading to an increase in positive charge and a reduce in hydrophobicity, which would contribute to the higher antimicrobial activity and safety (Table 1).

As anticipated, the antimicrobial activity of novel peptide 1018M increased by one time (Table 2), which may attribute to the enhanced positive charge (Table 1) and interaction ability to bacterial membranes. Interestingly, according to the results of molecular docking and HPLC, the ppGpp binding affinity of 1018M was also improved by the modification (Fig. 1, Additional file 1: Fig. S2), the possible explanation is that N terminal α-helix structure shortens the interaction distance between Arg2 and ppGpp. Subsequently, Arg2 formed more hydrogen bonds with ppGpp than Leu3 (Fig. 1).

Despite IDR-1018’s appealing therapeutic potential, its biological properties such as cytotoxicity and stability tend to limit their potential to emerge as effective drug candidates. Modifying these properties is a major approach to circumvent these issues. Compared to the parental peptide, the hemolysis of 1018M was significantly lower (Fig. 2b), which may resulted in the reduced hydrophobicity, thus leading to an improvement of intravenous administration safety. Additionally, the stability of the novel small peptide was enhanced as well because of the regular alpha helix structure (Fig. 2d–f). The temperature, pH and pepsase stabilities make 1018M have better processing, storage and internal environmental tolerance, while the trypsin resistance needs to be further explored.

Subsequently, the bactericidal mechanism of peptides was elucidated. The wrinkled cell wall, destroyed cell membrane, entered PI and blocked genomic DNA indicated that IDR-1018 can kill bacteria by interacting with bacteria cell wall and cytoplasmic membranes, permeabilizing the membrane, causing leakage of contents (Fig. 3), disrupting genomic DNA (Fig. 4), and eventually lead to cell death, which was also found in other antimicrobial peptides (Yang et al. 2017; Yang et al. 2019). The novel peptide 1018M basically shares the same bactericidal mechanism with IDR-1018. However, the damage of cell wall and membrane caused by 1018M is more severe as is shown in SEM and TEM images (Fig. 3). This may be related to the higher electrostatically and Trp-induced interaction of 1018M with cell wall and membrane, which was benefit greatly from the increased positive charge and Trp residues compared parental peptide IDR-1018.

Except for planktonic MRSA ATCC43300, its biofilm associated infections are characteristically chronic and frequently occur in hospitals (Periasamy et al. 2012). The efficiency of currently common antibiotics is still far from satisfactory against biofilm (Flemming et al. 2016). In contrast, various types of antibiotics are known to induce (p)ppGpp synthesis, leading to antibiotic adaptive resistance (Gilbert et al. 1990; Nguyen et al. 2011). In this study, the anti-biofilm effect of the novel peptide 1018M was evaluated for the first time. Significantly enhanced activity of 1018M against MRSA ATCC43300 biofilm was observed compared with IDR-1018 (Fig. 5). One of the possible explanations is that 1018M with higher ppGpp binding ability (Fig. 1), which triggered a greater decrease in bacterial ppGpp level than IDR-1018. ppGpp as a master regulator of almost all aspects of bacterial physiology including biofilm formation (Hobbs and Boraston 2019), supports the bacteria survival by either directly or indirectly stimulating the expression of genes involved in stress protection (Spira and Ospino 2020). Reduced ppGpp concentration weakened the tolerance capability of bacteria against stress conditions, thus leading to a formation inhibition of biofilm.

Furthermore, in *S. aureus*, the synthesize and/or hydrolyze of (p)ppGpp were catalyzed by RelA/SpoT homologue (RSH), RelP and RelQ enzymes with GTP as the precursor. (p)ppGpp effects on transcription and translation occur indirectly through the modulation of intracellular GTP levels (Hobbs and Boraston 2019). Transcriptional repressor CodY is activated by binding GTP. The depletion of GTP during the stringent response causes de‑repression of the CodY-regulated gene network involved in adaptation to stress (Hauryliuk et al. 2015). *SigB* is one of the genes that directly regulated by CodY (Lobel and Herskovits 2016). SigB is an essential regulator of *S. aureus* biofilm maturation. The lack of SigB activity results in increased *agr* expression, thus elevating extracellular protease levels and altering the murein hydrolase activity profile and high levels of *agr* expression have antibiofilm effects (Lauderdale et al. 2009). Additionally, different from CodY, RsbU could positively regulate sigB in a growth phase dependent manner (Lee et al. 2020).

Therefore, in this study, we focus on the transcriptional level of these ppGpp relevant genes after incubation by IDR-1018 and 1018M. The results showed that *RSH* and *relP*, *relQ* gene expression were significantly decreased after treatment with IDR-1018 and 1018M respectively, which suggested that the peptides could not only bind to ppGpp directly, but also regulate the gene expression of ppGpp synthetase (*RSH*, *relP* and *relQ*) and hydrolase (*RSH*) (Figs. 1 and 5) to reduce the biofilm formation ability of *S. aureus*. Additionally, the peptides could also significantly enhance and reduce the expression of *codY* and *rsbU* gene, then inhibited the transcriptional level of *sigB* gene, thus leading to a high-level expression of *AgrA* gene. Subsequently, staphylococcal protein A (SPA), which is encoded by the *spa* gene, could be strongly downregulated by *agr*. SPA is considered one of the central proteins involved in the adherence, colonization and biofilm formation of *S. aureus*. Moreover, among the adhesion factors, polysaccharide intercellular adhesion (PIA) which is encoded by *ica* operon is essential for biofilm formation in *staphylococci*. *ica* locus consists of icaADBC operon, *icaD* is the most prevalent biofilm forming gene, which performs principal role in the synthesis of exopolysaccharides (Goudarzi et al. 2019; Omidi et al. 2020). *icaD* gene could also be remarkably downregulated by these two peptides, especially by 1018M. The possible explanation is that regulation of peptides to gene expression is achieved by binding to bacterial genomic DNA directly (Fig. 4).

In brief, a novel peptide 1018M was designed in this study through molecular docking and amino acid substitution based on IDR-1018. 1018M was found to be more potent than IDR-1018 at destructing bacterial cell wall, permeating cell membrane and binding to ppGpp. Therefore, 1018M exhibited higher antimicrobial activity against MRSA and better effect on its biofilm. Additionally, the peptides could also exert their activity by disrupting genomic DNA and regulating the expression of ppGpp metabolism and biofilm forming related genes. This study paves a way for discovering novel peptide drugs in biofilm-related clinical applications.

## Supplementary Information

**Additional file 1: Table S1.** Design of ppGpp metabolism and biofilm formation related genes primer. **Fig. S1. **Predictions of IDR-1018 (A, B) and 1018M (C, D) tertiary structures. **Fig. S2. **Liquid chromatogram of peptides and ppGpp binding.

## Data Availability

My manuscript has data included as electronic supplementary material.
