# Supplementary material for: Design of a novel antimicrobial peptide 1018M targeted ppGpp to inhibit MRSA biofilm formation
Source: AMB Express. 2021 Mar 26;11:49. doi: 10.1186/s13568-021-01208-6 (PMC7997937; doi:10.1186/s13568-021-01208-6)
Supplement: Supplementary file 1 — Additional file 1: Table S1. Design of ppGpp metabolism and biofilm formation related genes primer. Fig. S1. Predictions of IDR-1018 (A, B) and 1018M (C, D) tertiary structures. Fig. S2. Liquid chromatogram of peptides and ppGpp binding. [file 13568_2021_1208_MOESM1_ESM.docx]

**AMB Express**

**Design of a novel antimicrobial peptide 1018M targeted ppGpp to inhibit MRSA biofilm formation**

Zhou Jiale ^1*^, Jiao Jian^2*^, Tan Xinyi ^1^, Xie Haoji^1^, Huang Xueqin^1^, Wang Xiao^**1^

1 Immunology Innovation Team, School of Medicine, Ningbo University, Ningbo, Zhejiang 315211, China

2 Department of biomedicine, Beijing City University, Beijing 100094

* They contributed equally to this work.

** Corresponding author.

(1) Postal address of all authors as:

Immunology Innovation Team, School of Medicine, Ningbo University, 818 Fenghua St., Jiangbei District, Ningbo, Zhejiang 315211, P. R. China

E-mail address: wangxiao@nbu.edu.cn

Phone: 0086-0574-87600763

ORCID: 0000-0002-5579-9759

**Table S1** Design of ppGpp metabolism and biofilm formation related genes primer

| Gene | Sequence (5' to 3') |
| --- | --- |
| *RSH-F* | TACATCGCACTGATTGCCCA |
| *RSH-R* | TTAAATTGCCGGCTGTCGAG |
| *relP-F* | TTGCCGGAATTCGCGTAGTA |
| *relP-R* | CGCGTTCTGCTAAAAAGACTGG |
| *relQ-F* | AGAAAGTGGTTACCGCTCGT |
| *relQ-R* | TCATCCGGATAAGCACCATCA |
| *rsbU-F* | CGCGTGAAGATGTGTTCAAGAC |
| *rsbU-R* | CTATCTCTTTATCGTGAACTTGAAG |
| *sigB-F* | GGTGCCATAAATAGATTCGATATGTCCTT |
| *sigB-R* | CTTTTGATTTCACCGATTACAGTAGGTACT |
| *spA-F* | GCGCAACACGATGAAGCTCAACAA |
| *spA-R* | ACGTTAGCACTTTGGCTTGGATCA |
| *CodY-F* | AAAGAAGCGCGCGATAAAGCTG |
| *CodY-R* | TGCGATTAATAGGCCTTCCGTACC |
| *AgrA-F* | AAGCATGACCCAGTTGGTAACA |
| *AgrA-R* | ATCCATCGCTGCAACTTTGTAGA |
| *icaD-F* | ATGGTCAAGCCCAGACAGAG |
| *icaD-R* | AGTATTTTCAATGTTTAAAGCAA |
| *16s rRNA-F* | GCTGCCCTTTGTATTGTC |
| *16s rRNA-R* | AGATGTTGGGTTAAGTCCC |

**Figures**

**
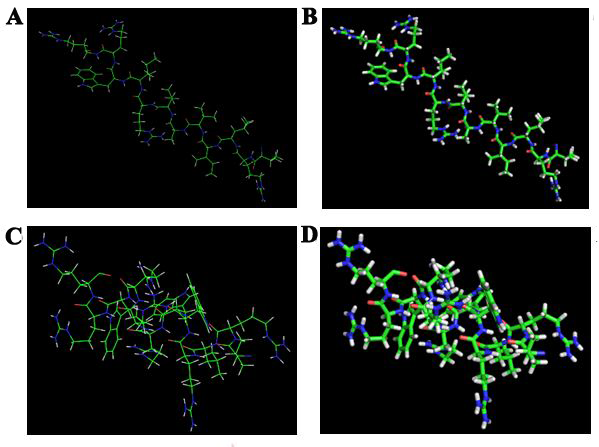
Figure 1**

**Fig. S1**

**Figure S1** Predictions of IDR-1018 (A, B) and 1018M (C, D) tertiary structures.

**Figure 2**

CK-1

CK-2

CK-3

ppGpp-1 retention time: 34.229 peak area: 31094

ppGpp-2 retention time: 34.263 peak area: 45985

ppGpp-3 retention time: 34.387 peak area: 27632

IDR-1018+ppGpp-1 retention time: 34.315 peak area: 19706

IDR-1018+ppGpp-2 retention time: 34.556 peak area: 18743

IDR-1018+ppGpp retention time: 34.096 peak area: 20720

1018M+ppGpp-1 retention time: 34.357 peak area: 13990

1018M+ppGpp-2 retention time: 34.210 peak area: 13997

1018M+ppGpp-3 retention time: 34.156 peak area: 14778

**Figure S2** Liquid chromatogram of peptides and ppGpp binding.
